# The relationship between cognitive perception of self‐concept and coping styles in heart failure patients

**DOI:** 10.1002/nop2.417

**Published:** 2019-11-16

**Authors:** Zahra Amouzeshi, Farzaneh Safajou, Tooba Kazemi, Sedigheh Kianfar

**Affiliations:** ^1^ Faculty of Nursing and Midwifery, Atherosclerosis and Coronary Artery Research Center Birjand University of Medical Sciences Birjand Iran; ^2^ Department of Medical Education Medical Education Development Research Center Isfahan University of Medical Sciences Isfahan Iran; ^3^ Student Research Committee School of Nursing & Midwifery Shahid Beheshti University of Medical Sciences Tehran Iran; ^4^ Ghaen Faculty of Nursing and Midwifery Birjand University of Medical Sciences Birjand Iran; ^5^ Atherosclerosis and Coronary Artery Research Center Department of Cardiology Birjand University of Medical Sciences Birjand Iran; ^6^ Department of Midwifery Birjand University of Medical Sciences Birjand Iran

**Keywords:** coping, heart failure, nurses, nursing, self‐concept

## Abstract

**Aim:**

To determine the relationship between self‐concept and coping styles in patients with heart failure in an Iranian population.

**Design:**

This study had a correlational design.

**Methods:**

In this study, 100 HF patients hospitalized in the CCU and cardiac ward of Vali‐Asr Hospital of Birjand, Iran were selected by convenience sampling method. Two validated and reliable questionnaires including Cognitive Perception of Self‐Concept and the Coping Styles questionnaires were completed by each patient.

**Results:**

There were significant associations between emotion‐oriented and threat to self‐concept in total and physical sensation and self‐consistency dimensions. Also, a significant association was observed between avoidance‐oriented and threat to self‐concept in total and body sensation dimension (*p* < .05). There was no significant association between the problem‐oriented component and the threat to self‐concept in total and none of its dimensions. The challenge to self‐concept and its dimensions were not significantly related to any component of coping styles.

## 1 INTRODUCTION

Cardiovascular diseases (CVDs) are the leading cause of mortality across the world. In 2012, the estimated mortality rate from CVDs was around 17.5 million, constituting 31% of all deaths. More than three‐quarters of deaths from CVDs occur in low‐ and middle‐income countries (WHO, 2015). Similar to other parts of Iran and the world, the most prevalent cause of death in Southern Khorasan is cardiovascular diseases (Kazemi & Nik, 2015).

Heart failure (HF) is a life‐threatening disease characterized by high mortality, recurrent hospitalization, poor quality of life and an often poor survival chance (Mahajan, Burman, & Hogarth, 2016). HF care costs are on the rise, and the costs arising from it have been estimated to override those of all malignancies and benign neoplasms altogether (Go et al., 2014). Early treatment can lead to improved quality of life, reduced hospitalization duration and lengthened lifetime (Ruppar, Cooper, Mehr, Delgado, & Dunbar‐Jacob, 2016).

Several CVD risk factors can be prevented (Dimsdale, 2008; Zeidan et al., 2016) among which are stress, depression and job and family tensions (Low, Salomon, & Matthews, 2009; Perk et al., 2012). When a stressor continues or a person develops a stressor in him/herself, negative effects appear on the heart's function. These negative effects can be moderated by changing behaviour and understanding and using stress management techniques (Chida & Steptoe, 2010; Dimsdale, 2008). Coping styles are certain strategies through which people respond to stressful situations (Nahlén Bose, Elfström, Björling, Persson, & Saboonchi, 2016). Folkman defines coping as a response to perceived stress (Folkman & Lazarus, 1980). In coping with stress, individuals use both emotion‐oriented and problem‐oriented coping styles. Emotion‐oriented styles include strategies such as avoidance, denial and behaviour disengagement; task‐oriented styles include active coping, problem‐solving and information‐seeking (Carver, Scheier, & Weintraub, 1989; Paukert, LeMaire, & Cully, 2009). Similar to stress, coping styles can meaningfully predict the occurrence of CVDs and affect complications and mortality from these diseases (Akbari, Mahmood Aliloo, & Aslanabadi, 2010; Doering et al., 2004; Nahlén Bose et al., 2016) so that increased levels of emotional style and stress increase risks of cardiac diseases (Akbari et al., 2010). Studies show that patients with HF who use maladaptive styles such as avoidance, denial would experience higher rates of fatigue, anxiety, anger and depression as well as a lower quality of life (Doering et al., 2004; Klein, Turvey, & Pies, 2007; MacMahon & Lip, 2002). In general, emotional styles are associated with lower general and mental health (Beasley, Thompson, & Davidson, 2003; McMahon et al., 2013).

## 2 BACKGROUND

A contributing factor to the selection of coping style is self‐concept. Self‐concept forms the main nucleus of any person's personality and is defined as a person's perception of his/her capabilities, appearance, emotions and values (Zhou, Wu, & Lin, 2012). A person's understanding of oneself is a multi‐dimensional issue, which is formed by a person's interaction and experience with the environment (Bong & Skaalvik, 2003). Positive self‐concept has a direct association with the selection of different dimensions of positive coping styles and health (Zhou et al., 2012) and can enhance satisfaction with life (Ritchie, Sedikides, Wildschut, Arndt, & Gidron, 2011). Individuals with positive self‐concept undergo lesser degrees of stress and tension than those with negative self‐concept (Kammeyer‐Mueller, Judge, & Scott, 2009). Results from Heydari et al's study on patients with HF in Iran showed that any threat to the components of self‐concept (body image, self‐ ideal, self‐ consistency, etc.) can lead to lower adherence to the therapeutic regimen on some patients. So that, if a person with HF takes the prescribed treatment regimen as a perceived threat, this threat can lead to the avoidance of coping style and thus non‐adherence to the prescribed health regimen (Heydari, Ahrari, & Vaghee, 2011).

Given the higher prevalence of CVDs, risk factors and mortality from them in Iran (Ebrahimi, Kazemi‐Bajestani, Ghayour‐Mobarhan, & Ferns, 2011; Forouzanfar et al., 2014) and because of increased economic burden and enormous costs to the health system, health researchers have tried to examine ways to reduce losses and improve the lives of patients and their compatibility with this disease. On the other hand, given that self‐concept is a psychological factor that involves a person's beliefs, feelings and attitude towards identity and personal values (Ahrari, Mohammadpour, Amouzeshi, & Agha‐Yousefi, 2014), it seems necessary to study this factor and its effects on coping style in different cultural communities. Although many studies on coping styles are conducted in various countries, it seems that culturally specific conditions in some countries are effective in coping styles (Knight & Sayegh, 2009). The results of these studies, therefore, cannot be generalized to other parts of the world. As a result, more research in other countries is necessary in this regard.

### 2.1 Aim

This study aimed to determine is there a relationship between self‐concept and coping styles in patients with HF?

### 2.2 Design

This is a correlational study, which was performed on 100 HF patients hospitalized in the CCU and cardiac wards of Vali‐e Asr Hospital in Birjand, Iran.

## 3 METHOD

Inclusion criteria included developing any form of HF, time passed over two months from HF diagnosis, full consciousness without a brain injury or mental disorders and age older than 20 years. The exclusion criteria were having a history of psychiatric and anxiety disorders. A pilot study was performed with 15 patients, to determine the sample size. A sample size of *N* = 100 was determined with consideration of the results of the pilot study, correlation formula, *α* = 0.05, power = 80 and *r* = 0.35 and an attrition rate of 25%.

After acquiring the required permissions from the Deputy of Research of Birjand University of Medical Sciences (ethical code: ir.bums.REC.1394.423) and verbal informed consent, those who were willing entered the study by convenience sampling method. The researcher filled out demographics form and Cognitive Perception of Self‐Concept and Coping Styles questionnaires via face‐to‐face interviews.

The Cognitive Perception of Self‐Concept questionnaire includes 57 items of which 31 are concerned with the threat to self‐concept and 26 are related to challenge to self‐concept. The responses are on a 4‐point Likert scale with each response receiving a score from 1–4. The total score of challenge to self‐concept is between 26–104 and that of the threat to self‐concept is 31–124. The questionnaire is used by Thomas (2007), and its validity is confirmed for HF patients (Thomas, 2007). The reliability and validity of the questionnaire are also confirmed in Iran (Ahrari, Heydari, & Vaghee, 2011).

Coping Styles Survey was originally developed by Endler and Parker in 1990 to evaluate how people face their problems. The questionnaire contains 48 items and three coping style domains, that is task‐oriented (problem‐oriented), which means control of emotions and step by step plan to solve the problem; emotion‐oriented coping where a person rather than focusing on the problem itself, focus on the emotion and instead of trying to solve the problem, strives to reduce negative emotions; and avoidance‐oriented coping style where the person avoids facing the problem. Responses are on a five‐point scale (number 1 = never; number 5 = always) (Endler & Parker, 1990), and its reliability and validity have been confirmed in Iran (Besharat, 2010). As the questionnaire held a relatively large number of items, the researcher completed the questionnaire at the time that the patients were mentally and physically in good condition. Whenever they were tired, the patients were asked to postpone completion of the questionnaire to sometime later.

### 3.1 Analysis

Data analysis was done by SPSS 16 software (IBM Incorporation). Quantitative variables normality was determined in the Kolmogorov–Smirnov test. Categorical variables were analysed using the chi‐square analysis. Pearson correlation coefficient, Mann–Whitney, *t* test, one‐way ANOVA and Kruskal–Wallis were used for comparison of continuous variables. The significance level is considered at *p* < .05.

### 3.2 Ethics

The code of ethical approval for this study is *ir.bums.REC.1394.423.*

## 4 RESULTS

Among the participants, 63% were men and 37% were women. Most were in the 51–60 age range. Other demographic characteristics are presented in Table 1. Scores related to the self‐concept of patients (threat and challenge) is given in Tables 2 and 3.

**Table 1:** Demographic characteristics of participants

Demographic variable	*N* (%)
Gender	
Male	63 (63)
Female	37 (37)
Age	
≤50	15 (15)
51–60	31 (31)
61–70	29 (29)
>70	25 (25)
Education level	
No formal educational	41 (41)
Primary	38 (38)
High school and above	21 (21)
History of other diseases	
Yes	43 (43)
No	57 (57)
Health insurance	
Yes	95 (95)
No	5 (5)
Income level	
Less than enough	56 (56)
Enough	44 (44)
More than enough	0 (0)

**Table 2:** Patients’ scores for Threat to self‐concept

Threat to self‐concept dimensions	Minimum	Maximum	Mean (*SD*)
Body sensation	8	24	14.01 (3.50)
Body image	8	20	13.02 (2.29)
Self‐consistency	14	27	18.44 (2.64)
Self‐ideal	6	17	9.45 (2.1)
Spiritual self	2	6	3.44 (1.26)
Total	45	79	58.36 (7.42)

**Table 3:** Patients’ scores for challenge to self‐concept

Challenge to self‐concept dimensions	Minimum	Maximum	Mean (*SD*)
Body sensation	6	16	11.06 (1.73)
Body image	10	24	17.96 (2.51)
Self‐consistency	10	22	15.24 (2.41)
Self‐ideal	12	24	18.18 (2.93)
Spiritual self	6	11	9.00 (1.44)
Total	51	92	71.44 (9.01)

The results indicate that there is a significantly positive association between emotion‐oriented style and the threat to self‐concept in total and between body sensation and self‐consistency (*p* < .05). There was also a positive significant association between avoidance‐oriented style and the threat to self‐concept as well as its body sensation dimension (*p* < .05). No association was found between problem‐oriented style and the threat to self‐concept in total or any of its dimensions (*p* > .05) (Table 4). The challenge to self‐concept and its dimensions were not significantly related to any of the coping styles’ dimensions (Table 5).

**Table 4:** The relationship between threat to self‐concept, its dimensions and components of coping styles

Threat to self‐concept	Coping styles		
	Emotion‐oriented	Avoidance‐oriented	Problem‐oriented
Body sensation	*r* = 0.34	*r* = 0.30	*r* = 0.07
	*p* = .001	*p* = .002	*p* = .52
Body image	*r* = 0.14	*r* = 0.02	*r* = 0.04
	*p* = .16	*p* = .81	*p* = .73
Self‐consistency	*r* = 0.27	*r* = 0.15	*r* = 0.002
	*p* = .006	*p* = .14	*p* = .98
Self‐ideal	*r* = 0.14	*r* = 0.13	*r* = 0.10
	*p* = .16	*p* = .20	*p* = .31
Spiritual self	*r* = 0.04	*r* = 0.08	*r* = 0.05
	*p* = .71	*p* = .41	*p* = .66
Total	*r* = 0.35	*r* = 0.23	*r* = 0.04
	*p < *.001	*p* = .02	*p* = .69

**Table 5:** The relationship between challenge to self‐concept, its dimensions and components of coping styles

Challenge to self‐concept	Coping styles		
	Emotion‐oriented	Avoidance‐oriented	Problem‐oriented
Body sensation	*r* = 0.08	*r* = 0.10	*r* = 0.07
	*p* = .41	*p* = .32	*p* = .42
Body image	*r* = 0.07	*r* = 0.09	*r* = 0.08
	*p* = .48	*p* = .35	*p* = .48
Self‐consistency	*r* = 0.13	*r* = 0.16	*r* = 0.17
	*p* = .19	*p* = .11	*p* = .08
Self‐ideal	*r* = 0.02	*r* = 0.03	*r* = 0.10
	*p* = .86	*p* = .78	*p* = .31
Spiritual self	*r* = 0.19	*r* = 0.08	*r* = 0.04
	*p* = .06	*p* = .45	*p* = .73
Total	*r* = 0.06	*r* = 0.03	*r* = 0.11
	*p* = .54	*p* = .80	*p* = .28

Comparison of mean scores of the threat to self‐concept and challenge and their dimensions in the patients in terms of demographic characteristics indicated that challenge to self‐concept was significantly higher in its self‐ideal dimension among 51–60 age group than 70 plus year‐old patients. Moreover, the threat to self‐concept mean score of body image was significantly higher in illiterate patients than elementary level literate ones. On the other hand, challenge to self‐concept mean score of body sensation was significantly greater in elementary level literate patients than illiterate ones.

## 5 DISCUSSION

In this study, there was a significantly positive association between emotion‐oriented and avoidance‐oriented components and the total score of the threat to self‐concepts. When a person interprets a stimulator as a threat, the stimulator changes into stress and the person applies emotional responses such as situation shift, denial or avoidance as well as emotional responses to relieve stress (Carpenter, 2008).

The results from Heydari et al's study (2011) showed that the threat to the components of self‐concept can induce emotion‐oriented responses in patients with HF, thus a reduced adherence to the recommended health regimen (Heydari et al., 2011). Thomas also believes that a health regimen may be considered as a threat to self‐concept to patients suffering from HF, thus causing emotional responses in them and reduced adherence to the regimen (Thomas, 2007). Similar findings are reported in Sung (2011) and Kammeyer‐Mueller et al. (2009) and Ahrari et al. (2014) (Ahrari et al., 2014; Kammeyer‐Mueller et al., 2009; Sung, 2011).

In this study, there was no significant association between the problem‐oriented component and threat to self‐concept in total or its dimensions. Perhaps because of this reason is that when a person interprets a stimulator as a threat, she/he will use emotional and avoidant responses (Heydari et al., 2011). Akbari's study showed that CHD patients compared with healthy individuals have higher stress levels and tend more to use an emotion‐oriented coping style than a problem‐oriented one in the face of stress (Akbari et al., 2010). However, Klein mentions that although the adoption of maladaptive coping styles such as denial or self‐blame is associated with lower life quality and increased depression, the patients with HF tend more to use adaptive coping styles such as active coping, acceptance and planning. This shows that these patients strive to cope more effectively with their problems (Klein et al., 2007).

With regard to body sensation, there was a significant association between the threat to this dimension of self‐concept and emotion‐oriented and avoidance‐oriented coping styles. This finding is compatible with findings from Heydari et al's study. One reason why threats and challenges are associated with body sensation and adherence to a health regimen, Heydari et al explain, lies with the different bodily experiences that patients with HF have in association with their diseases. This can influence the kind of response and compliance with the situation (Heydari et al., 2011).

In the present study, challenge to self‐concept and its dimensions did not show a statistically significant association with coping styles, a finding that was in contradiction with the results of previous studies (Sung, 2011; Thomas, 2007). For example, Heydari et al's study shows that if patients with HF consider the prescribed therapeutic regimen as a challenge, coping behaviours such as problem‐solving will appear that lead to greater adherence to the regimen (Heydari et al., 2011). Coping with an event as a threat or challenge depends on the type of event. An individual's personality and coping resources may also affect his/her perspective and analysis of the encountered situation. In other words, coping as the threat or challenge can have a reflective potential depending on the interaction between the individual and the surrounding environment. If the person receives support from the environment and also seeks for supportive resources, his/her coping can shift from a negative perspective to a positive perception (Zhan, 2000).

## 6 LIMITATIONS

Among the limitations of the study is that the responses provided by the participants might have been influenced by their psychological and mental conditions outside the researchers’ control. Furthermore, coping styles and cognitive perception are changing processes in individuals. This can also be regarded as a limitation of the present study.

## 7 CONCLUSION

According to the results of this study, it can be understood that in the context of perceiving oneself, the threat to self‐concept dimension can affect coping styles in patients with HF. Through identification of these aspects and nursing interventions such as support, counselling and education, the patients can be assisted in their coping with this disease such that they can use better (problem‐solving) coping styles in stressful situations.

## CONFLICT OF INTEREST

There are no conflicts of interest associated with this study.
